# Supplementary material for: Neprilysin 4 controls acrosome structure and male fertility in Drosophila melanogaster
Source: Commun Biol. 2025 Nov 22;8:1701. doi: 10.1038/s42003-025-09186-2 (PMC12658024; doi:10.1038/s42003-025-09186-2)
Supplement: Supplementary file 2 — Supplementary Information [file 42003_2025_9186_MOESM2_ESM.pdf]

Supplementary Material

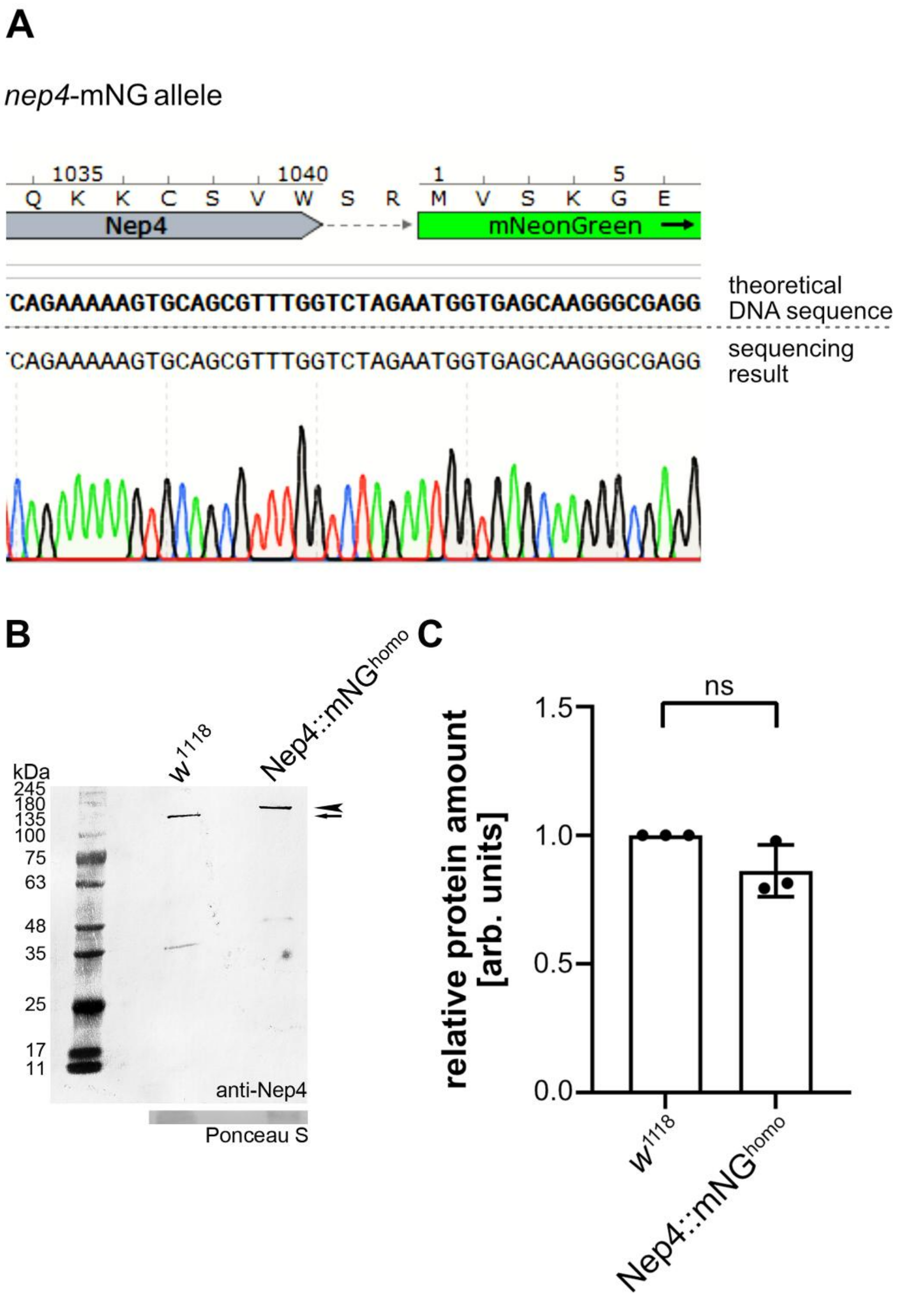

Supplementary Figure 1: The Nep4::mNeonGreen fusion protein exhibits proper stability and molecular mass. (A) In-frame insertion of the mNeonGreen coding sequence at the endogenous locus of *nep4* is confirmed by sequencing genomic DNA isolated from Nep4::mNG^{homo} animals. The lower panel depicts the corresponding sequence electropherogram at the fusion site. (B) Testes from homozygous Nep4::mNG animals (Nep4::mNG^{homo}) were analyzed by Western blot probed with anti-Nep4 antibodies. Testes from w^{1118} animals were used as a control. A band corresponding to Nep4 is visible with expected molecular mass (119.6 kDa, arrow) in protein extracts of control testes. In corresponding extracts from Nep4::mNG^{homo} animals, this band is replaced by a band with a higher molecular mass, presumably representing the Nep4::mNG fusion protein (expected molecular mass: 146.3 kDa, arrowhead). The lower panel depicts the loading control (Ponceau S). (C) Quantification of (B). The amount of Nep4 protein is not significantly affected by the fusion of the mNeonGreen tag. All signal intensity values were normalized to the respective loading controls (Ponceau S). ns = not significant (paired t-test, two-tailed). Each dot represents one individual biological replicate.

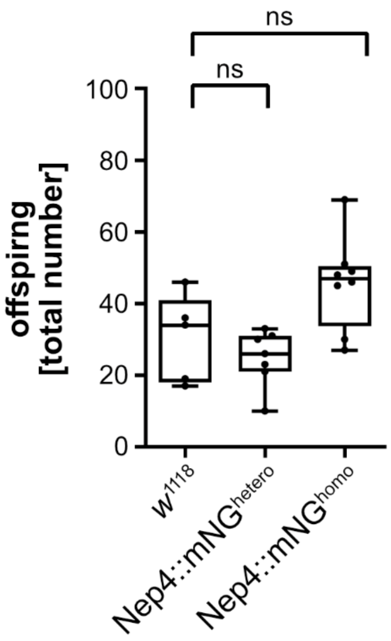

Supplementary Figure 2: Female Nep4::mNG flies are fertile. Virgin females of the indicated genotypes were mated to wildtype males. Offspring were counted after 18 days. The number of offspring from heterozygous and homozygous Nep4::mNG females (Nep4::mNG^{hetero}; Nep4::mNG^{homo}) is comparable to that of control females (w^{1118}; one-way ANOVA followed by Tukey's Multiple Comparison Test; ns = not significant). Each dot represents one individual experiment. At least five individual biological replicates were analyzed for each genotype.

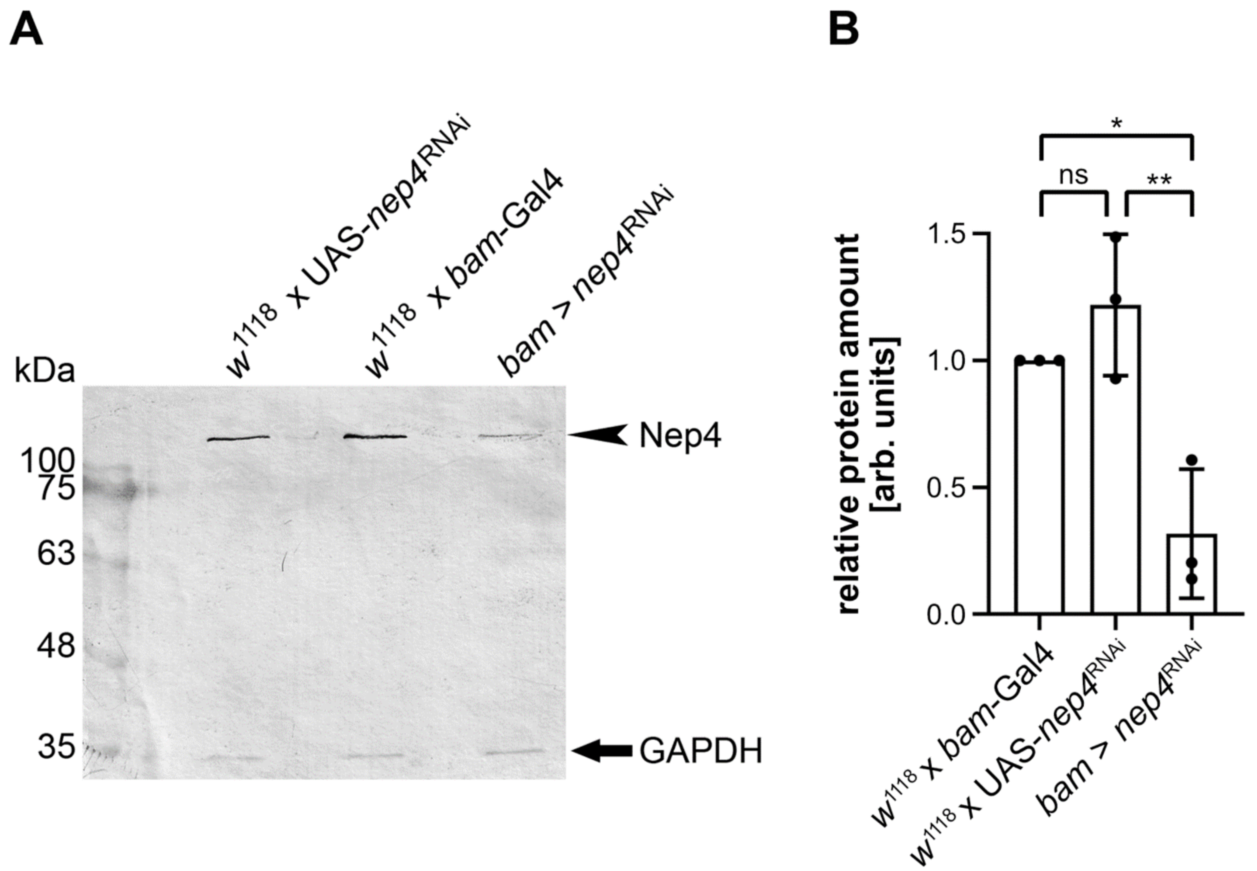

Supplementary Figure 3: Knockdown of *nep4* is efficient in testes. (A) Testes from control (w^{1118} x *bam*-Gal4 or w^{1118} x UAS *nep4*^{RNAi}) as well as knockdown animals (*bam* > *nep4*^{RNAi}) were analyzed by Western blot probed with anti-Nep4 antibodies. A band corresponding to Nep4 is visible with expected molecular mass (119.6 kDa, arrowhead) in protein extracts of all testes analyzed. The signal intensity appears to be weaker in the knockdown testes compared to the controls. GAPDH was detected as a loading control (arrow). The blot shown is representative of all three biological replicates performed. For each replicate, 40 testes were isolated per genotype. (B) Quantification of (A). Nep4 protein amount is significantly reduced in *nep4* RNAi testes, relative to both controls. All signal intensity values were normalized to the respective loading controls (GAPDH). Asterisks indicate statistically significant differences (*$p < 0.05$, **$p < 0.01$, Repeated Measures (RM) one-way ANOVA followed by Tukey's Multiple Comparison Test; ns = not significant). Each dot represents one individual biological replicate. Animals were reared at 27 °C.

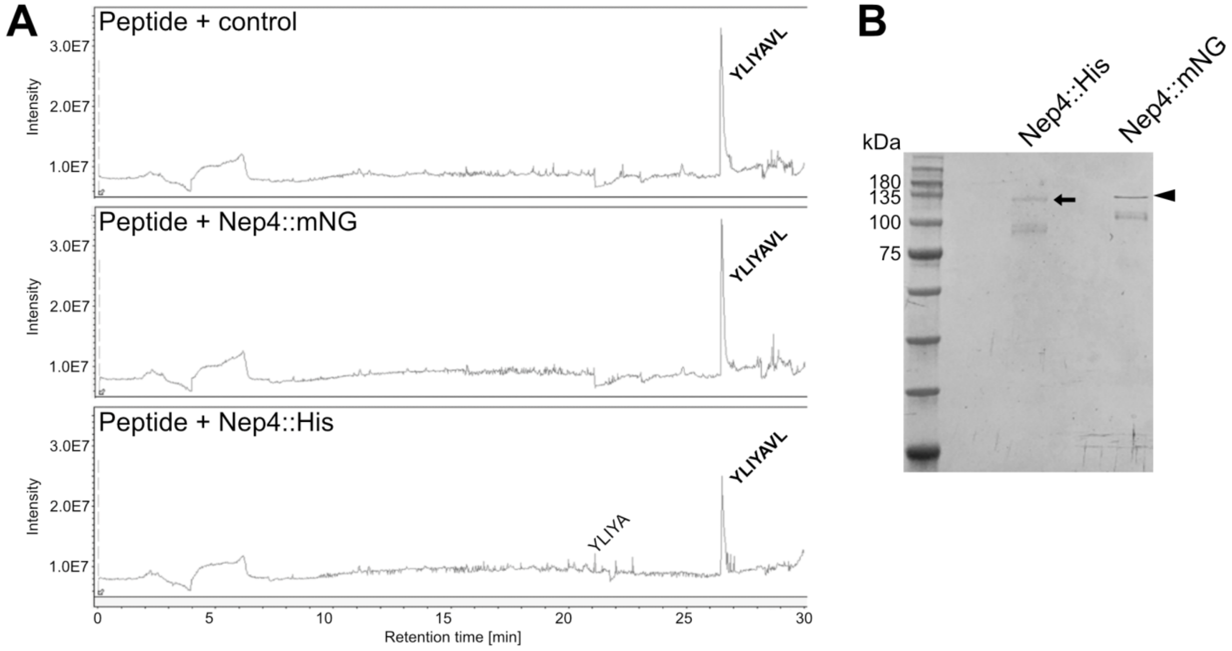

Supplementary Figure 4: mNeonGreen-tagged Nep4 is catalytically inactive. (A) Depicted are total ion chromatograms of the luminal part of Sarcolamban A (YLIYAVL) incubated with either mNeonGreen-tagged Nep4 (Peptide + Nep4::mNG), His-tagged Nep4 (Peptide + Nep4::His), or a control preparation without Nep4 (Peptide + control). Full-length peptides (bold) are detected under all applied experimental conditions. Specific cleavage fragments (YLIYA) are detected only after addition of the His-tagged Nep4 construct. (B) Coomassie stained SDS-PAGE analysis of purified His-tagged Nep4 (Nep4::His) or mNeonGreen-tagged Nep4 (Nep4::mNG). Both constructs migrate close to their expected molecular mass (Nep4::His: 115 kDa, arrow; Nep4::mNG: 141 kDa, arrowhead). Purity and stability of both constructs appear similar.

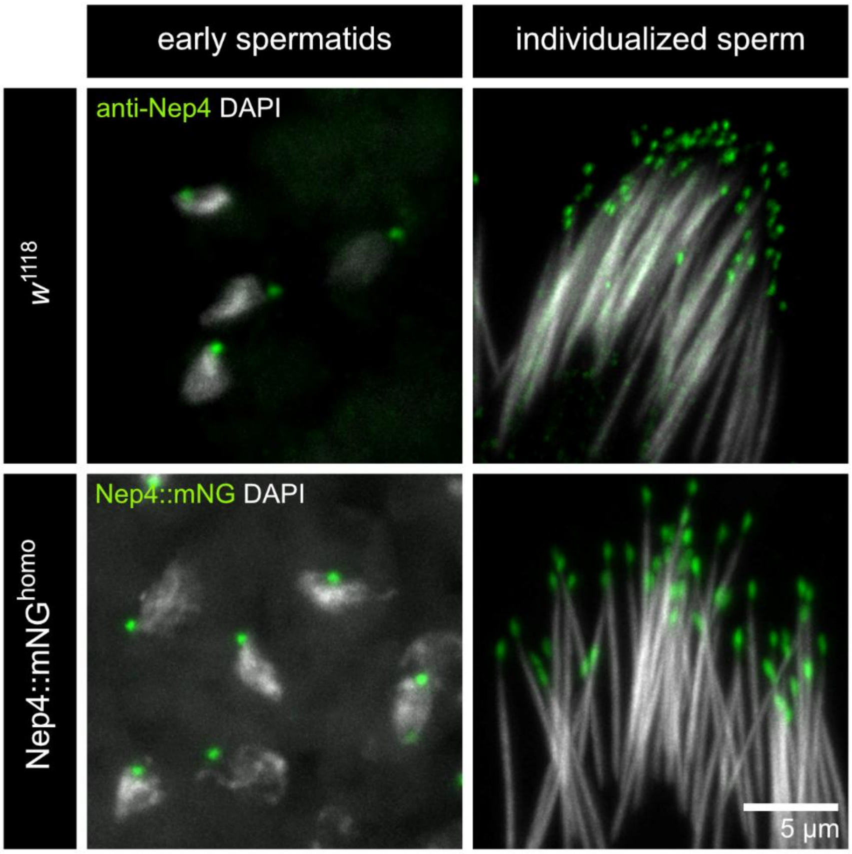

Supplementary Figure 5: The subcellular localization of Nep4::mNG reflects the localization of the endogenous protein. Spermatids/individualized sperm isolated from 1-2 days old males are shown. The nuclei are visualized by DAPI staining (gray). Untagged Nep4 protein in control samples (w^{1118}) is visualized by anti-Nep4 antibodies detecting the endogenous protein. mNeonGreen-tagged Nep4 in Nep4::mNG^{homo} animals is detected via its fluorescence signal. The localization pattern of the untagged and the tagged protein (green) in early spermatids and in individualized sperm is virtually identical in both samples, which indicates a wildtype-like subcellular localization of the Nep4::mNG fusion protein.

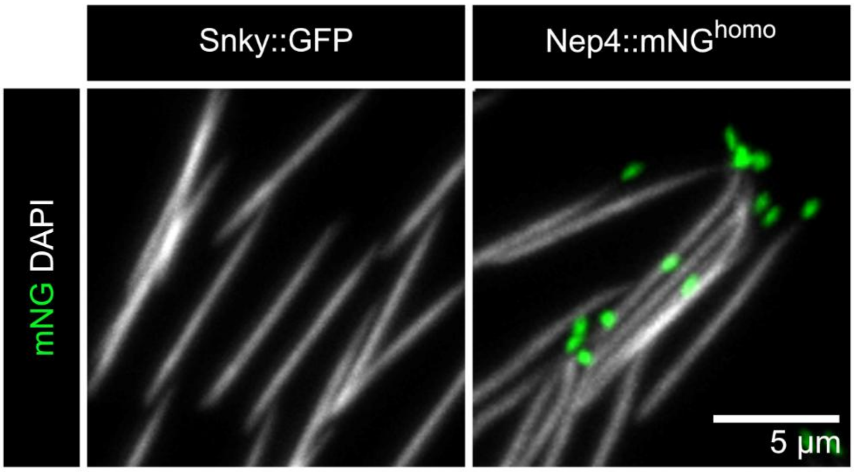

Supplementary Figure 6: Snky::GFP signal is not detected by the applied mNeonGreen acquisition parameters. Sperm dissected from 1-2 days old males of the indicated genotypes are shown. The nuclei are visualized by DAPI staining (gray). To exclude the possibility that the Snky::GFP signal is detected by the applied mNeonGreen acquisition parameters, both fluorophores were imaged using the same parameters. Due to the considerably brighter mNeonGreen signal, relative to the GFP signal, the latter was not detected under these conditions.

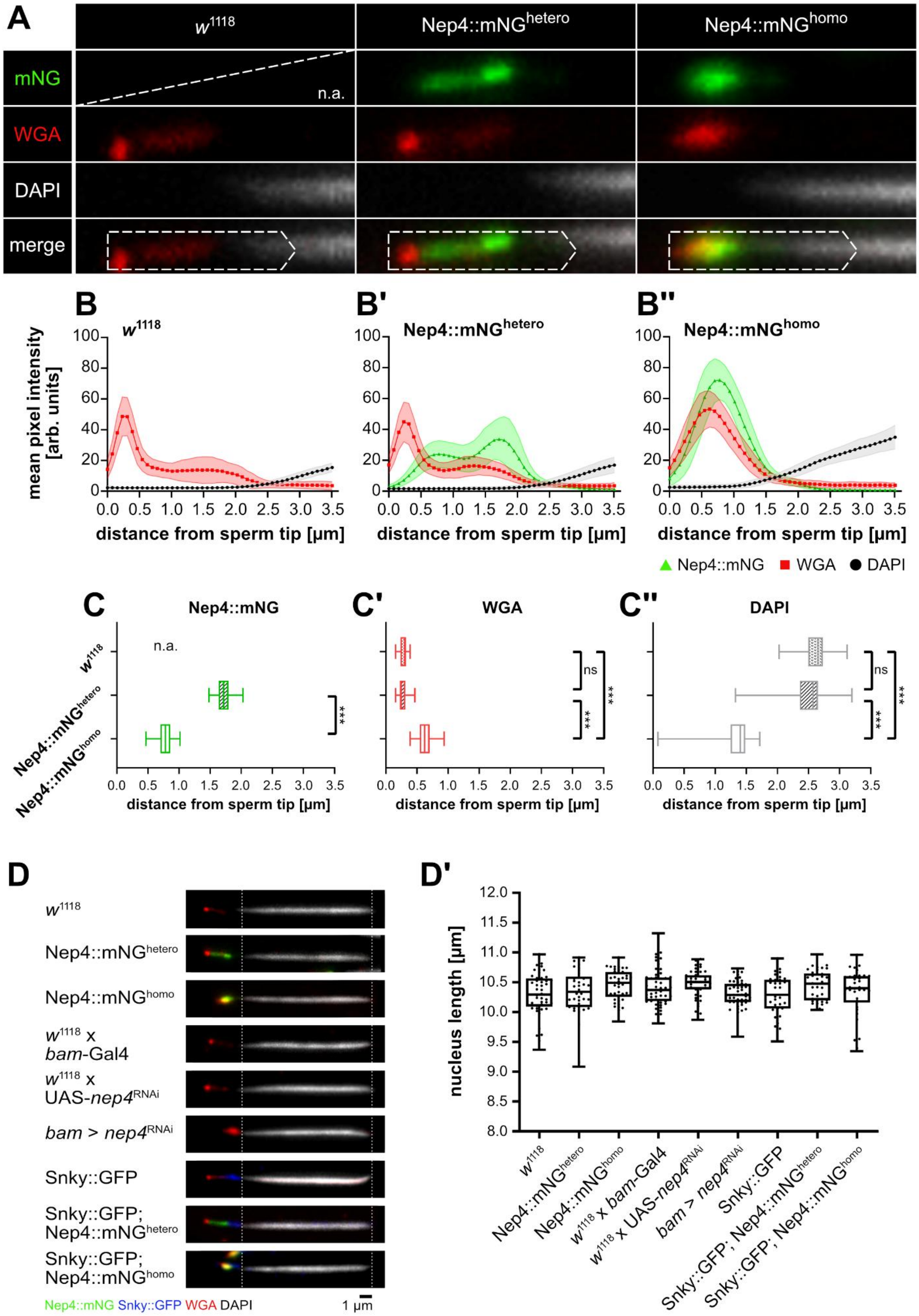

A
w1118
Nep4::mNGhetero
Nep4::mNGhomo
mNG
WGA
DAPI
merge
n.a.
B
B'
B''
mean pixel intensity [arb. units]
distance from sperm tip [µm]
Nep4::mNG
WGA
DAPI
C
C'
C''
n.a.
ns
***
D
D'
w1118 x bam-Gal4
w1118 x UAS-nep4RNAi
bam > nep4RNAi
Snky::GFP
Snky::GFP; Nep4::mNGhetero
Snky::GFP; Nep4::mNGhomo
Nep4::mNG Snky::GFP WGA DAPI
1 µm
nucleus length [µm]

Supplementary Figure 7: Nep4::mNG levels affect sperm tip partitioning. The sperm from 1-2 days old flies of the indicated genotypes were dissected and stained with WGA (red) and DAPI (gray). The Nep4::mNG signal (green) is preserved during the staining procedure. Sperm of control (w^{1118}), Nep4::mNG^{hetero}, and Nep4::mNG^{homo} animals are analyzed. (A) The distal part of the sperm tip is depicted in single channel images and a merged image for each genotype. Dashed open arrows (0.7 x 3.5 µm) indicate the regions analyzed by pixel intensity measurements. (B-B'') The mean values of the individual pixel intensities ± SD are shown. For each genotype, at least 50 individual sperm cells isolated from the testes of at least five different animals were analyzed. While in control and Nep4::mNG^{hetero} animals all signals show a well distinguishable distribution, the characteristic pattern is lost in Nep4::mNG^{homo} animals. (C-C'') Characteristic parameters of the pixel intensity measurements are analyzed. The individual distances of the maximum intensities of the Nep4::mNG (C) and WGA signals (C') from the sperm tip are shown. In addition, the distance from the sperm tip at which the DAPI signal surpasses a predefined threshold is depicted (C''). Asterisks indicate statistically significant deviations from controls (w^{1118}; ***$p < 0.001$; one-way ANOVA followed by Tukey's Multiple Comparison Test; ns = not significant). n.a. = not applicable. (D) Representative images of the entire sperm nuclei of all genotypes analyzed in this study. Dashed lines indicate the borders of the individual nuclei. (D') Quantification of (D). The mean values of the sperm nucleus lengths ± SD are shown. For each genotype, a subset of at least 30 individual sperm cells isolated from the testes of at least five different animals was analyzed. No significant differences in sperm length are present (one-way ANOVA followed by Tukey's Multiple Comparison Test).

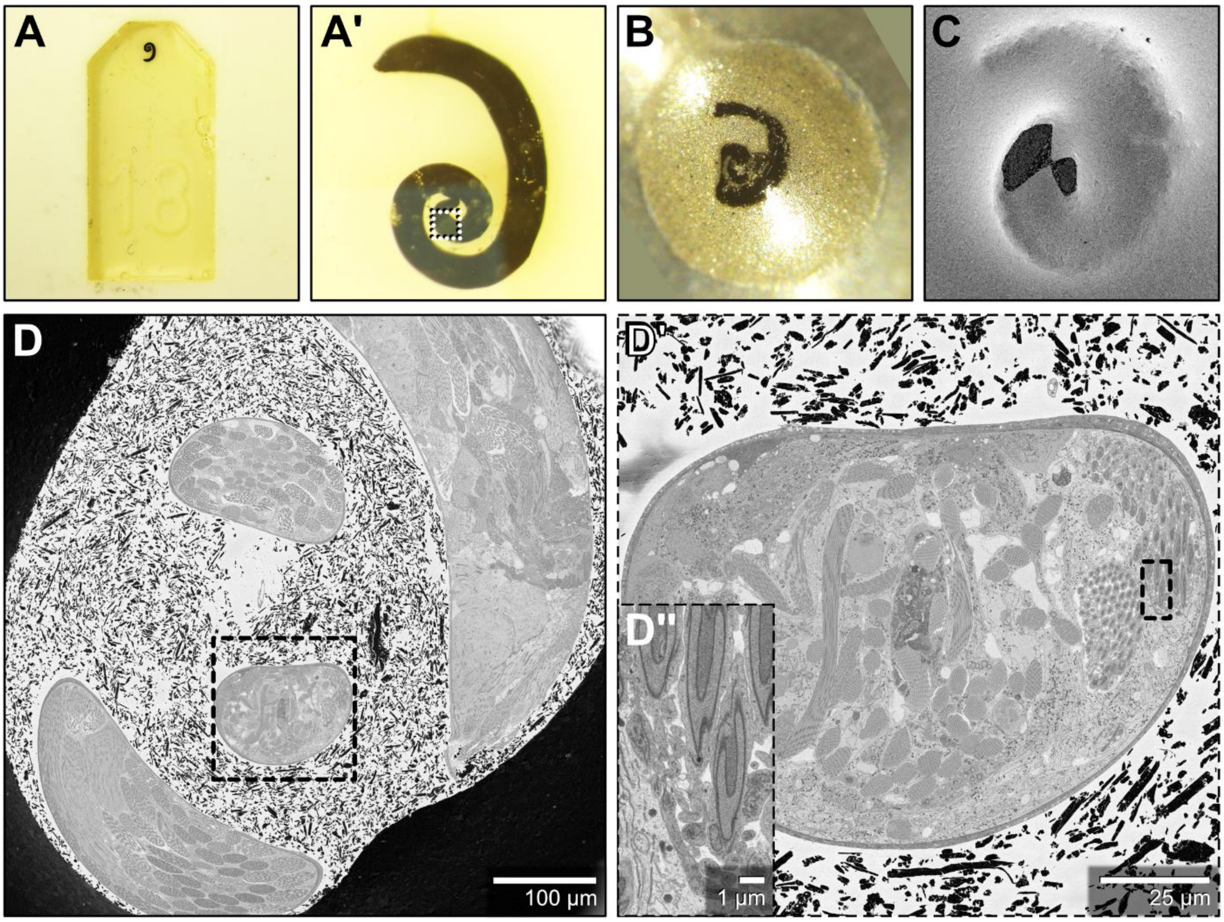

Supplementary Figure 8: Minimal embedding of whole testes facilitates precise targeting of sub-organellar regions in SBF-SEM. The testes of 1–2-day old flies were dissected and then prepared for electron microscopy. (A, A') Isolated testes were ROTO-stained, dehydrated, and infiltrated with resin. (B) After infiltration, samples were placed on absorbent paper to remove excess resin and subsequently transferred to a sample rivet using conductive silver adhesive. (C) This minimal resin approach improves the identification and localization of specific sub-regions within organs or tissues when utilizing secondary electron detection mode. (D) The integrated ultramicrotome of the SBF-SEM enables precise targeting of areas near the basal end of the testis in a slice-and-view manner. The dashed box highlights a region that corresponds approximately to that indicated in A'. (D') High-resolution overview scans of the indicated regions in D facilitate the reliable localization of the anterior tip of mature elongated spermatid bundles. (D'') The inset shows a representative area of interest, indicated by the dotted box in D', revealing a cross-section of spermatids that displays nuclei and part of the acrosomal region.

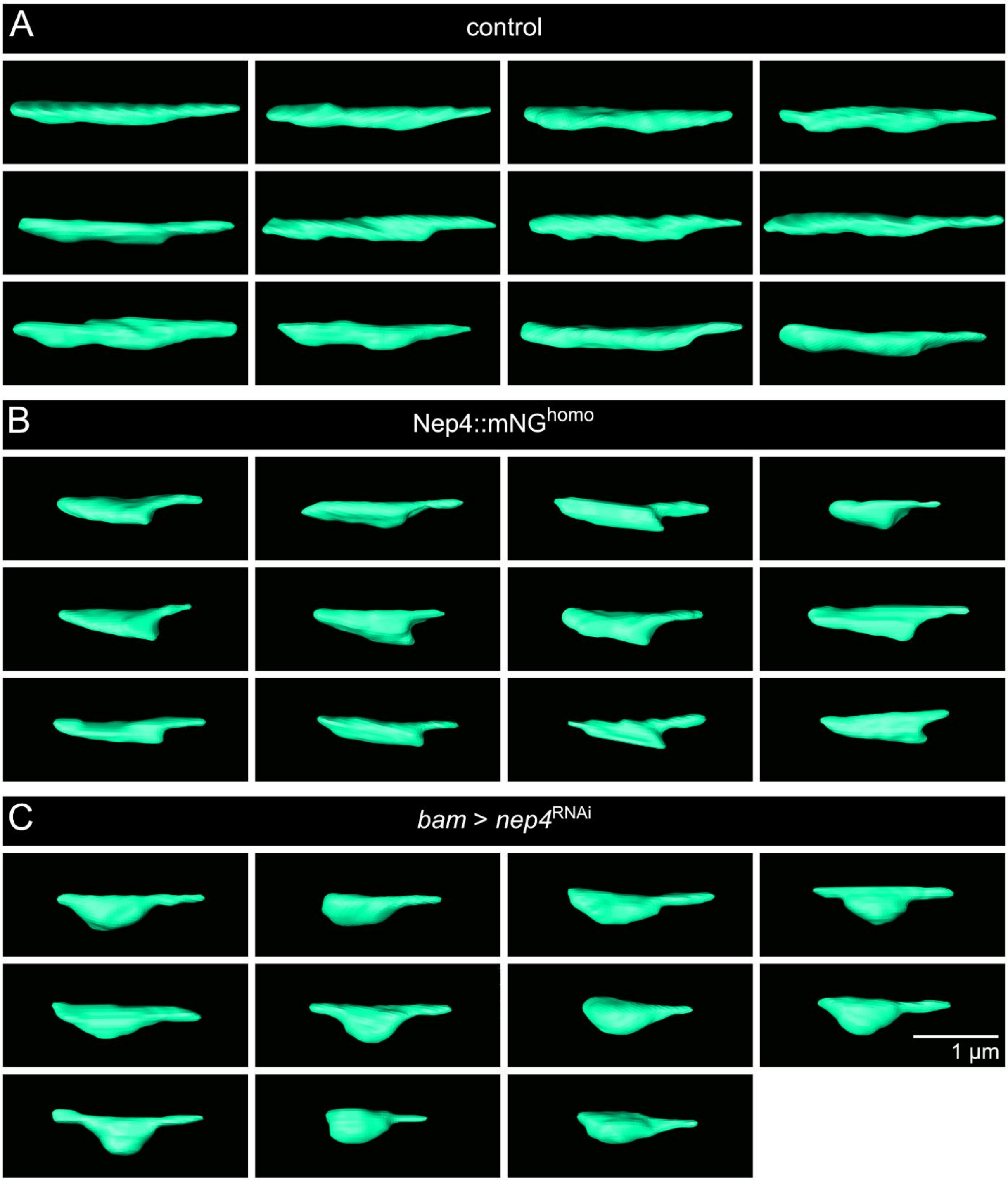

Supplementary Figure 9: Impaired Nep4 function results in abnormal acrosome structure. Testes isolated from 1-2 days old flies of the indicated genotypes were analyzed by serial block-face scanning electron microscopy (SBF-SEM). Volume rendered images based on the SBF-SEM data of all analyzed acrosomes are depicted. A considerably impaired morphology is visible for acrosomes isolated from Nep4::mNG^{homo} (B) and *nep4*^{RNAi} knockdown animals (*bam* > *nep4*^{RNAi}, C), compared to control acrosomes (*w*^{1118} x UAS-*nep4*^{RNAi}, A). Both Nep4::mNG^{homo} and *nep4*^{RNAi} acrosomes appear shorter and wider than the control organelles. The depicted 3D reconstructions were used to calculate acrosome length and volume, as shown in Figure 6.

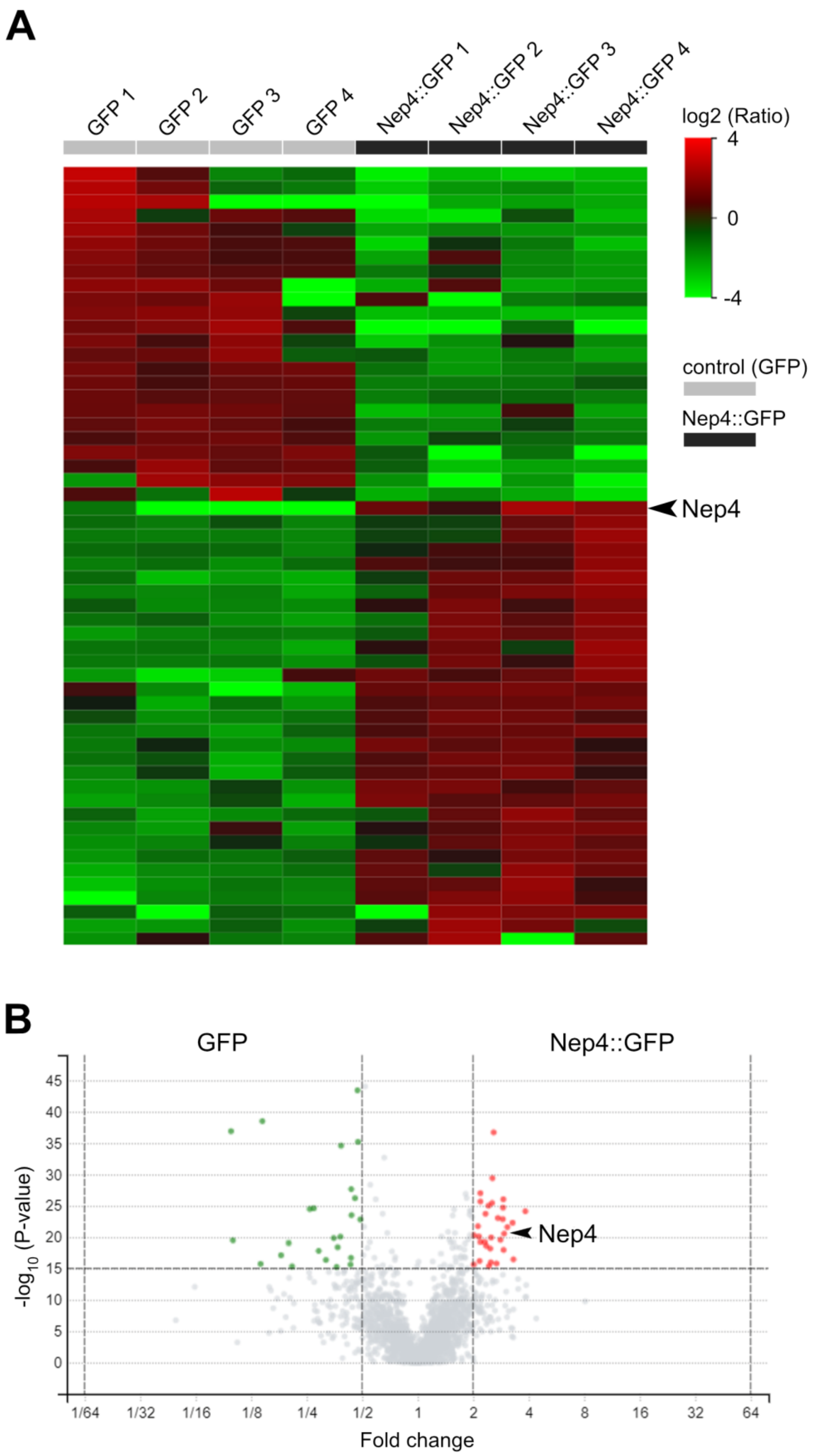

Supplementary Figure 10: Nep4::GFP does not interact with cytoskeletal components. (A) Heatmap of GFP pull-down replicates performed in 1-week-old control male flies expressing GFP in sperm cells (*bam* > GFP, gray bar) or 1-week-old male flies expressing a Nep4::GFP fusion protein in sperm cells (*bam* > Nep4::GFP, black bar). The Nep4::GFP fusion protein (bait) is indicated (arrowhead). (B) Volcano plot of the same experiment depicting proteins that co-precipitate with a Nep4::GFP fusion protein expressed in sperm cells (*bam* > Nep4::GFP, red dots) or with a GFP control expressed in sperm cells (*bam* > GFP, green dots). The Nep4::GFP fusion protein (bait) is indicated (arrowhead). All significantly enriched proteins are depicted in Supplementary Table 1.

Supplementary Data 2: Results of testes-specific co-precipitation analyses using Nep4::GFP as bait. Depicted are proteins that exhibit significantly increased amounts in pull-down fractions isolated from 1-week-old male flies expressing a Nep4::GFP fusion protein as bait in sperm cells (*bam* > Nep4::GFP), relative to 1-week-old control male flies expressing free GFP as a control in sperm cells (*bam* > GFP). Proteins that significantly co-precipitate with Nep4::GFP are shown in bold ($p < 0.05$, one-way ANOVA).

Supplementary Video 1: TEM-tomogram of the acrosome region in sperm isolated from heterozygous Nep4::mNG flies as depicted in Figure 3A. Sperm isolated from heterozygous Nep4::mNG flies were prepared for on-section CLEM and a high-resolution tomogram of an area of interest was recorded. The acrosome exhibits an elongated shape.

Supplementary Video 2: TEM-tomogram of the acrosome region in sperm isolated from homozygous Nep4::mNG flies as depicted in Figure 3A. Sperm isolated from homozygous Nep4::mNG flies were prepared for on-section CLEM and a high-resolution tomogram of an area of interest was recorded. The acrosome exhibits a rather spheroid shape.

Supplementary Video 3: Reconstruction of control spermatids and their acrosomal region via SBF-SEM as depicted in Figure 6A. Image acquisition was performed with 4 x 4 x 40 nm voxel resolution. The slice series were further used for segmentation, reconstruction and measurement of the acrosomal region. Movie shows the respective 2D-slice series through a bundle of spermatids, digital sections through the acrosomal region with the acrosomal structure segmented, then a representative 3D volume of a spermatid containing the acrosome (turquoise), nucleus (grey), axoneme (magenta) and the surrounding plasma membrane (transparent white outline). Acquired volume: 33 x 41 x 11 µm.

Supplementary Video 4: Reconstruction of Nep4::mNG^{homo} spermatids and their acrosomal region via SBF-SEM as depicted in Figure 6A. Image acquisition was performed with 4 x 4 x 40 nm voxel resolution. The slice series were further used for segmentation, reconstruction and measurement of the acrosomal region. Movie shows the respective 2D-slice series through a bundle of spermatids, digital sections through the acrosomal region with the acrosomal structure segmented, then a representative 3D volume of a spermatid containing the acrosome (turquoise), nucleus (grey), axoneme (magenta) and the surrounding plasma membrane (transparent white outline). Acquired volume: 49 x 66 x 12 µm.

Supplementary Video 5: Reconstruction of *bam* > *nep4*^{RNAi} spermatids and their acrosomal region via SBF-SEM as depicted in Figure 6A. Image acquisition was performed with 4 x 4 x 40 nm voxel resolution. The slice series were further used for segmentation, reconstruction and measurement of the acrosomal region. Movie shows the respective 2D-slice series through a bundle of spermatids, digital sections through the acrosomal region with the acrosomal structure segmented, then a representative 3D volume of a spermatid containing the acrosome (turquoise), nucleus (grey), axoneme (magenta) and the surrounding plasma membrane (transparent white outline). Acquired volume: 34 x 50 x 17 µm.
