# Supplementary material for: Neprilysin 4 controls acrosome structure and male fertility in Drosophila melanogaster
Source: Commun Biol. 2025 Nov 22;8:1701. doi: 10.1038/s42003-025-09186-2 (PMC12658024; doi:10.1038/s42003-025-09186-2)
Supplement: Supplementary file 3 — Description of Additional Supplementary files [file 42003_2025_9186_MOESM3_ESM.pdf]

Description of Additional Supplementary files

File name: Supplementary Data 1
Description: The source data behind the graphs in the paper

File name: Supplementary Data 2
Description: List of proteins that interact with Nep4 in testes, identified by pull-down assays.

File name: Supplementary Video 1
Description: TEM-tomogram of the acrosome region in sperm isolated from heterozygous Nep4::mNG flies as depicted in Figure 3A.

File name: Supplementary Video 2
Description: TEM-tomogram of the acrosome region in sperm isolated from homozygous Nep4::mNG flies as depicted in Figure 3A.

File name: Supplementary Video 3
Description: Reconstruction of control spermatids and their acrosomal region via SBF-SEM as depicted in Figure 6A.

File name: Supplementary Video 4
Description: Reconstruction of Nep4::mNG^{homo} spermatids and their acrosomal region via SBF-SEM as depicted in Figure 6A.

File name: Supplementary Video 5
Description: Reconstruction of *bam* > *nep4*^{RNAi} spermatids and their acrosomal region via SBF-SEM as depicted in Figure 6A.
